# Fatigue on Waking, Insomnia, and Workplace Relationship Problems May Help to Detect Suicidal Ideation among New Middle-Aged Primary Care Patients: A 6-Month Prospective Study in Japan

**DOI:** 10.3390/ijerph20085547

**Published:** 2023-04-17

**Authors:** Megumi Fujieda, Katsuhisa Uchida, Shinichiro Ikebe, Akihiro Kimura, Masashi Kimura, Toshiaki Watanabe, Hisako Sakamoto, Teruaki Matsumoto, Naohisa Uchimura

**Affiliations:** 1 Department of Neuropsychiatry, Kurume University School of Medicine, 67 Asahi-machi, Kurume 830-0011, Fukuoka, Japan; 2 Department of Environmental Medicine, Kurume University School of Medicine, 67 Asahi-machi, Kurume 830-0011, Fukuoka, Japan; 3 Department of Healthcare Management, College of Healthcare Management, 960-4 Takayanagi, Setaka-machi, Miyama 835-0018, Fukuoka, Japan; 4 Mental Health and Welfare Center of Shizuoka Prefectural Government, 2-20 Ariake-cho, Suruga-ku, Shizuoka-shi 422-8031, Shizuoka, Japan; 5 Fuji Medical Association, 2850 Denbo, Fuji 417-0061, Shizuoka, Japan; 6 Shizuoka General Hospital, 4-27-1 Kitaando, Aoi-ku, Shizuoka-shi 420-8527, Shizuoka, Japan

**Keywords:** diagnosis, epidemiology, fatigue, insomnia, internal medicine, suicide prevention campaign

## Abstract

Signs of suicidal depression often go undetected in primary care settings. This study explored predictive factors for depression with suicidal ideation (DSI) among middle-aged primary care patients at 6 months after an initial clinic visit. New patients aged 35–64 years were recruited from internal medicine clinics in Japan. Baseline characteristics were elicited using self-administered and physician questionnaires. DSI was evaluated using the Zung Self-Rating Depression Scale and the Profile of Mood States at enrollment and 6 months later. Multiple logistic regression analysis was conducted to calculate adjusted odds ratios for DSI. Sensitivity, specificity, and likelihood ratios for associated factors were calculated. Among 387 patients, 13 (3.4%) were assessed as having DSI at 6 months. Adjusted for sex, age, and related factors, significant odds ratios for DSI were observed for “fatigue on waking ≥1/month” (7.90, 95% confidence intervals: 1.06–58.7), “fatigue on waking ≥1/week” (6.79, 1.02–45.1), “poor sleep status” (8.19, 1.05–63.8), and “relationship problems in the workplace” (4.24, 1.00–17.9). Fatigue on waking, sleep status, and workplace relationship problems may help predict DSI in primary care. Because the sample size in this investigation was small, further studies with larger samples are needed to confirm our findings.

## 1. Introduction

Most suicidal patients go undiagnosed. Diagnosing suicidal patients’ mental health problems in a busy clinical practice with a short consultation period is challenging. The mean duration of visits to primary care physicians was reported to be 16.3 min in the United States [1,2]. Approximately 85% of medical consultations were reported to be less than 20 min in Japan [3,4]. If individuals at high risk of suicide can be identified by asking simple questions, physicians may be able to manage a patient’s prognosis more effectively, particularly through treatment or referral. If physicians fail to control patients’ high-risk conditions, they may refer them to mental health specialists before imminent risk of suicide. For patients who are at high risk of suicide but have no complaints about mental instability, the procedure of detecting and referring is frequently difficult until the patient is in imminent danger of suicide. Before developing evident suicidal thoughts, physicians should know more about each patient’s suicide risk condition.

According to epidemiological studies of the general population, the 1-year prevalence of suicidal ideation among adults in the United States ranges from 2.3% to 5.6% [5,6]. Suicidal patients are likely to visit primary care clinics before suicide occurs [7]. According to one study, 45% and 77% of suicide patients visited a primary care clinic 1 month and 1 year before suicide, respectively [8]. However, primary care doctors have difficulty identifying their patients’ suicidal ideation [9,10,11,12,13]. In one study, reviewing inpatients’ clinical charts revealed that 78% of suicide victims denied having suicidal thoughts and intent in their last communication before suicide [14].

Four major components of primary care suicide interventions—namely, educating practitioners, screening for suicide risk and/or mood disturbance, managing depression symptoms, and assessing and managing suicide risk—were reported [15].

Regarding the first component, educating practitioners, educational programs alone are insufficient for preventing suicide [16,17]. With regard to the second component, screening for suicide risk and/or mood disturbance, it can be difficult to conduct routine screening for suicidal ideation, which includes items such as hopelessness, thoughts of killing oneself, and suicide attempts in patients who seek care primarily for physical concerns, particularly at the time of initial medical consultation [18].

Regarding the third component, managing depression symptoms, recognizing depression and beginning treatment are crucial. However, in situations of primary care, depression is frequently overlooked. One meta-analysis found that the prevalence of depression in primary care was 19.5%, but almost half of depressive patients were not identified in primary care [19]. Thus, we previously reported the importance of asking about insomnia at initial medical consultations in primary care because very high specificity and sensitivity were observed for depression in combinations of insomniac subtypes [20].

As for the fourth component, assessing and managing suicide risk, assessing for the presence of suicide risk factors and managing suicide risk may help to prevent suicide. Suicidal behavior is rare in the absence of current symptoms of major psychiatric disorders [21]. Depression has been reported in 50% to 87% of completed suicides [22]. Approximately 60% of depressed patients experienced suicidal ideation during their current depressive episode [23,24]. The relative risks for suicidal attempts during depressive episodes were 7.54 to 33.5 [25,26]. Insomnia has been reported as one of the risk factors of suicidal behavior and is a treatable condition [21]. However, insomnia and the extent of risk of suicidal ideation in primary care settings has not been thoroughly investigated. The association between insomnia and suicidal ideation has been reported among psychiatric patients [27], community residents, the general population [28], patients who died by suicide [29], and other specific populations (e.g., the veteran population) [30]. Few studies conducted among non-psychiatric outpatients have reported an association between insomnia and suicidal ideation [31,32]. Moreover, these investigations were cross-sectional and did not include middle-aged patients at primary care clinics in the community [31,32].

A PubMed search using the keywords “morning fatigue and suicide”, “fatigue on waking and suicide”, and “morning drowsy and suicide” found no articles. School non-attendance due to difficulty waking up is increasing in Japan [33]. Some studies have investigated difficulties in waking up; however, we found no research investigating fatigue on waking. Furthermore, to the best of our knowledge, no studies have investigated the association between fatigue on waking and suicidal ideation. This proposed association developed from clinical observation. According to Nakai, to prevent suicide in depressed patients, it is important to address their discomfort at waking up in the morning [34]. Thus, the association between fatigue on waking and depression with suicidal ideation (DSI) was investigated in this study.

Inoue et al., conducted an investigation among 17,390 males and 2933 females employed at nine Japanese factories [35]. They reported that males who had high and moderate interpersonal conflict showed significantly higher adjusted odds ratios for depression than those who had low interpersonal conflict (OR = 2.00–4.88). In Japan, the number of suicides among men, middle-aged people, and employees is high [36,37,38,39]; therefore, relationship problems in the workplace are thought to be worth investigating as a potential factor associated with DSI.

In an initial medical consultation and/or in time-constrained circumstances, simple inquiries about issues such as insomnia, fatigue on waking, and relationship problems in the workplace are not difficult to ask about before moving on to more in-depth discussions about suicide and depression. To the best of our knowledge, the associations between the patient’s condition, including insomnia, at the initial medical consultation and the new onset of DSI several months after the initial medical consultation have not been investigated. The present prospective study was conducted to address this issue. New patients aged 35–64 years attending primary care clinics were followed for 6 months. If physicians are able to identify a patient’s risk of DSI, early diagnosis, treatment, and/or referral may be offered. Otherwise, patients at imminent risk of suicide might not be accepted immediately because of inaccessibility of psychiatrists [40] or patients’ anosodiaphoria [41,42].

From 1998 to 2011, more than 30,000 people died by suicide in Japan. To address this, in 2006, the Basic Law on Suicide Countermeasures was enacted. Since that year, a suicide prevention campaign, the Fuji Model Project, has been carried out in Shizuoka Prefecture with the aim of reducing the high suicide rate among middle-aged males [43].

A structure of collaboration between primary care practices and psychiatric clinics (hospitals) was formed during the campaign. In Shizuoka Prefecture, general public education is also carried out. The campaign contains messages about insomnia such as “Dad, are you getting enough sleep?”, “Talk with your primary care physician” and “Sleeplessness lasting more than 2 weeks is a sign of depression” that were broadcast on TV. Leaflets containing these phrases were distributed through pharmacies, alcohol shops, and local government-related institutions. The campaign was expanded and conducted nationwide.

The number of suicides in Japan in 2021 was 21,007 [39]. In the general population, 22.9 suicides per 100,000 people were reported. The suicide rates among men and women were 16.8 and 11.0 per 100,000 people, respectively. Suicides among men numbered 13,939, which is 66.4% of total suicides. Suicide rates according to age group were 3618 (17.2%) for the 50–59 years group, 3575 (17.0%) for the 40–49 years group, 3009 (14.3%) for the 70–79 years group, and 2637 (12.6%) for the 60–69 years group. According to occupation, suicide rates were 11,639 (55.4%) for the unemployed, 6692 (31.9%) for employees, 1298 (6.2%) for self-employed or family workers, and 1031 (4.9%) for students and others. Prior to the COVID-19 outbreak up until 2021, the number of suicides per 100,000 in the population significantly increased among females and people aged 10–19 years and 20–29 years of age [36,37,38]. The suicide rates in 2018, 2019, 2020, and 2021 were 10.1, 9.4, 10.9, and 11.0 for females, 5.0, 5.3, 5.9, and 7.0 for people aged 10–19 years, and 17.7, 17.1, 16.8, and 19.8 for people aged 20–29 years, respectively. The number of female suicides increased from 6550 in 2018, 6091 in 2019, 7026 in 2020 to 7068 in 2021. Therefore, the actual numbers of suicides in Japan are higher among men, middle-aged people, unemployed people, and employed people.

The Basic Law on Suicide Countermeasures calls for early identification of suicidal individuals in medical facilities as well as the collaboration of doctors specializing in physical and mental health. This regulation mandated that all physicians should detect suicidal ideation in patients and refer them to the appropriate mental health institutions.

This investigation was conducted to evaluate the concept of the Fuji Model Project, which could potentially be carried out in any country, regardless of its level of economic development. Additionally, the general public and non-medical professionals can aid in the prevention of suicide. The findings of the current study may assist doctors in identifying patients who are at risk for suicide and treating the condition before it becomes serious, especially in a time-constrained busy professional practice setting.

## 2. Materials and Methods

### 2.1. Study Subjects

**Figure 1:**
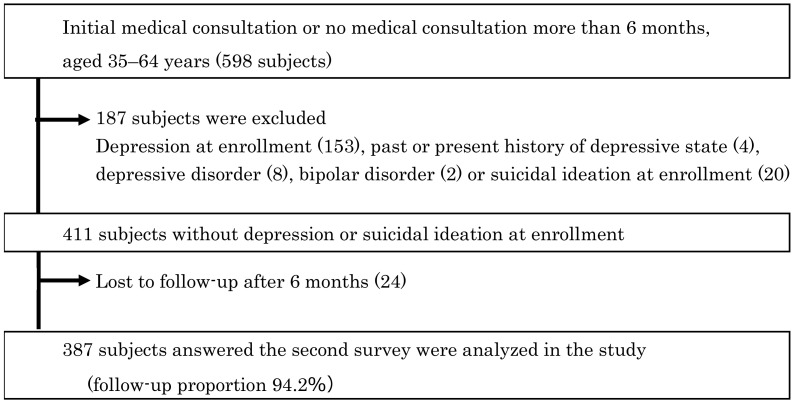
Flow chart of enrollment of the study subjects.

Subjects were new patients aged 35–64 years, recruited from three internal medicine clinics in Fuji, Shizuoka Prefecture, Japan, from 10 May 2011 to 24 May 2012. The definition of a new patient was someone visiting a clinic for the first time or who had not visited the clinic in more than 6 months. Ineligibility criteria for study subjects were body temperature ≥37.5 °C, clear externally caused injury, visual impairment, or auditory difficulties, because these conditions were considered to be likely to interfere with individuals’ ability to fill out the questionnaires. Eligible patients were consecutively asked to participate in the study at each clinic. A total of 600 subjects (200 from each clinic) were enrolled in the study. One subject participated in this study twice and another subject was found to be 65.4 years old (i.e., over 65 years). These two subjects were excluded. Of 598 enrollees, 187 were excluded. Of these, 153 were assessed as depressed at enrollment, 14 enrollees without depression at enrollment had a diagnosed history of a depressive state (four subjects), depressive disorder (eight subjects), or bipolar disorder (two subjects), and 20 subjects without depression at enrollment or the diagnosed history mentioned above had suicidal ideation at enrollment (Figure 1). Of the remaining 411 subjects, 387 participated in a follow-up investigation at 6 months after enrollment.

The Ethics Committee of Kurume University approved the study protocol (No. 10286). This study was conducted in accordance with the Declaration of Helsinki (October 2008). Written informed consent was obtained from all subjects before participation.

### 2.2. Information Collection

The details of information collection for this study have been previously described [20]. Briefly, subjects were asked to fill out three self-administered, structured questionnaires to provide physical, mental, and environmental information at the time of enrollment before medical consultation. Consulting physicians also filled out a structured questionnaire. The self-administered questionnaires were not shown to the physicians to avoid influencing the physicians’ diagnoses.

Information from the first self-administered questionnaire included sex, age, exercise habits, major life events, relationship problems in the workplace, family discord, alcohol and smoking history (including amount per day), educational background, occupation, marital status, family history of psychiatric disorders, psychiatric illnesses, and other underlying diseases. Feeling of isolation from other family members was measured using a visual analogue scale consisting of a 100 mm horizontal line; this ranged from no feeling of isolation to a feeling of extreme isolation. The Japanese version of the Pittsburgh Sleep Quality Index (PSQI) was included to assess sleep status [44,45]. In addition, the Japanese version of the Zung Self-Rating Depression Scale (SDS) and the Profile of Mood States (POMS) were used to assess depression [46,47,48,49,50,51,52]. The questionnaire for physicians collected information on diagnosis, chief complaints, prescriptions, and referral to a mental health specialist.

At 6 months after enrollment, subjects were asked to fill out a questionnaire inquiring about workplace absences and/or sick leave during the past 6 months, reasons and diagnoses for any absenteeism, medical consultation at psychosomatic medicine and/or psychiatric clinics and/or hospitals, and the name of any medical institutions where consultation for psychiatric problems took place. The SDS and POMS were also re-administered.

Reliability and validity tests of the Japanese version of the PSQI, SDS, and POMS were previously conducted in Japan [44,47,50]. However, we also tested their reliability and validity in this study. The Cronbach’s alpha for the seven component scores of the PSQI global score was 0.577 among 387 subjects [44]. This is because depressive subjects were removed from the 598 enrolled subjects. This result is consistent with the previous investigation, which showed that lower Cronbach’s alphas were observed among control subjects (0.43), while higher Cronbach’s alphas were observed among subjects with major depression (0.72) and subjects with primary insomnia (0.74) [44]. In the present study, among 387 subjects, the standardized Cronbach’s alpha for 15 component items in the depression–dejection subscale of POMS at 6 months and for 20 items in the SDS at 6 months were 0.837 and 0.796, respectively [47,50]. This is much greater than the suggested value of 0.70 [53]. The mean, median, and standard deviation of the depression–dejection subscale score of POMS were 8.9, 9.0, and 4.5 among subjects without DSI and 17.0, 16.0, and 6.1 among subjects with DSI, respectively, with a *p* value in the Wilcoxon rank sum test <0.0001. The mean, median, and standard deviation of the SDS were 36.5, 36.0, and 6.8 among subjects without DSI and 50.0, 49.0, and 7.9 among subjects with DSI, respectively, with a *p* value <0.0001. For the PSQI, the mean, median, and standard deviation were 4.6, 4.0, and 2.2 among subjects without DSI and 6.9, 7.0, and 3.2 among subjects with DSI, respectively, with a *p* value of 0.007.

### 2.3. Definition of Poor Sleep and Fatigue on Waking

The PSQI is a self-administered questionnaire designed to assess quantitative and qualitative aspects of sleep during the previous month [44,45]. Individuals with a global score of ≥6 are defined as poor sleepers, whereas those with scores of ≤5 are defined as good sleepers. Both global scores and scores for individual items, such as sleep quality, frequency of difficulty falling asleep, and frequency of waking in the middle of the night or early morning, were used for the analyses. The Japanese version of the Epworth Sleepiness Scale was also used to measure drowsiness in the daytime [54,55].

In addition, the frequency of fatigue on waking was measured by the question “Do you have difficulty getting up because of fatigue?” Subjects were asked to choose the most appropriate answer, as follows: “none”, “less than once per month”, “once or more per month but less than once per week”, or “once or more per week”. This question is referred to as “fatigue on waking” in the following text and tables.

### 2.4. Case Definition

Subjects who were assessed as being in a depressed state by both the SDS and POMS were defined as having depression. We used the SDS translated into Japanese; this scale contains 20 items, scored from 1 to 4 [48]. The total score was multiplied by 1.25 to obtain the SDS index [48]. An SDS index value of 50, equivalent to a raw score of 40, was used as a morbidity cutoff score [48]. The POMS assesses six mood states. The depression–dejection subscale score is calculated from responses to 15 items, with a score range from 0 to 60 [51]. The mean plus the standard deviation of a so-called healthy adult population was used as the cutoff score for depressive mood state (cutoff scores are 18.3, 16.3, 16.9, and 13.7 for males and 17.4, 16.3, 14.2, and 12.8 for females aged 35–39, 40–49, 50–59, and ≥60 years, respectively) [51].

Suicidal ideation was measured using the question “I feel that others would be better off if I were dead”, which is included in the SDS [56]. Subjects answering “some of the time”, “a good part of the time”, or “most or all of the time” were defined as having suicidal ideation.

### 2.5. Data Analyses

Subjects with and without depression accompanied by suicidal ideation were compared using the chi-square test or Fisher’s exact test. For continuous variables, the Wilcoxon rank sum test was used. Sensitivity, specificity, positive and negative predictive value, positive likelihood ratios (LR+), and negative likelihood ratios (LR−) of associated factors for DSI were calculated [57,58]. The formula “pretest odds × likelihood ratio = posttest odds” was used. Pretest odds were derived using the formula “pretest odds = prevalence/(1 − prevalence).” Posttest probability was derived using the formula “posttest probability = posttest odds/(1 + posttest odds)” [58]. Pretest probability and posttest probability correspond to pre-inquiry probability and post-inquiry probability, respectively, in the following text.

To assess predictive factors for DSI, adjusted odds ratios (ORs) and their 95% confidence intervals (95% CIs) were calculated using logistic regression. All analyses except for likelihood ratios and their CIs were conducted using SAS version 9.4 (SAS Institute Inc., Cary, NC, USA). A *p* value of less than 0.05 (two-sided) was considered significant.

Correlations of fatigue on waking and factors related to sleep status (global and individual PSQI score, global and individual Epworth Sleepiness Scale score (sleepiness during daytime)), alcohol consumption, feeling of isolation, economic difficulty, and total SDS score at enrollment and 6 months after enrollment were calculated.

Explanatory variables of the model were sex, age, regular exercise habits, problems involving care of the family, death of a family member, relationship problems in the workplace, cigarette smoking, history of psychiatric disorder excluding dementia in parent(s) or child (children), educational background, mean alcohol consumption per day [59], feeling of isolation from other family members, and fatigue on waking (Model 1) or PSQI global score (Model 2). The stepwise method with default *p* values of 0.05 to enter and remove was used to determine the final model. Regular exercise habits, problems involving care of the family, death of a family member, relationship problems in the workplace, cigarette smoking, history of psychiatric disorder excluding dementia in parent(s) or child (children), mean alcohol consumption per day and fatigue on waking were selected through the stepwise method. Feeling of isolation from other family members and PSQI global score were included in the final models because of statistical significance in the univariate analysis. Demographic variables of sex, age, and educational background were included in the final models irrespective of stepwise selection and statistical significance in the univariate analysis.

Mean alcohol consumption per day was calculated using grams of ethanol and a standard conversion table for alcoholic beverages (i.e., beer was assumed to be 5% ethanol, wine 12%, sake 15%, shochu 25%, and whiskey 40%) [59].

Power analysis after the investigation was conducted using an SAS power procedure. Two-category variables of relationship problems in the workplace (no and yes), fatigue on waking divided into two categories (“less than once per month” and “once or more per month”), and the global score of PSQI divided into two categories of (“5 or less [no sleep disturbance]” and “6 or more [sleep disturbance]”), (“6 or less” and “7 or more”), and (“9 or less” and “10 or more”) were used for power analysis. The frequencies and their ORs for DSI were also used for power analysis.

## 3. Results

Of the 411 subjects eligible for follow-up, 387 (94.2%) completed the second survey at 6 months after enrollment. Data from the 387 subjects were analyzed. The median age of subjects was 49.0 years with a range of 35.1–64.4 years (1st quartile: 41.4, 3rd quartile: 56.4), and 48.6% were male. At the initial medical consultation, respiratory system diseases, including the common cold, defined through the International Statistical Classification of Diseases and Related Health Problems, Tenth Revision (ICD-10) code J00–J99, were most frequent (40.1%). Diseases occurring with high frequency were those of the musculoskeletal system and connective tissue (M00–M99) (12.9%), endocrine, nutritional, and metabolic diseases (E00–E90) (10.9%), circulatory system diseases (I00–I99) (10.3%), and digestive system diseases (K00–K93) (7.0%). No subjects had psychotic symptoms or dementia at the initial medical consultation.

DSI was observed in 13 subjects (3.4%). Table 1 shows the characteristics of all subjects and the adjusted ORs for DSI. Significantly increased ORs were observed for fatigue on waking once or more per month but less than once per week (OR: 7.90 [95% CI: 1.06–58.7]) and once or more per week (OR: 6.79 [1.02–45.1]) in Model 1. Increased ORs were observed for total PSQI scores of 6, 7–9, and ≥10; the OR for scores of ≥10 was significant (OR: 8.19 [1.05–63.8]) in Model 2, with a significant dose–response relationship (*p* for trend: 0.038). Significant ORs were observed for relationship problems in the workplace (OR: 4.24 [1.00–17.9] and OR: 4.84 [1.14–20.6]) in Model 1 and Model 2, respectively.

Conversely, no significant adjusted ORs were observed for difficulty falling asleep within 30 min, waking in the middle of the night or early morning, quality of sleep and average length of sleep during the past month. After adjustment for variables in Model 1, except for fatigue on waking, the OR for difficulty falling asleep within 30 min less than once per week was 2.15 (95% CI, 0.42–11.1), that for 1–2 times per week was 0.57 (0.03–12.8), and that for ≥3 times per week was 5.47 (0.71–42.0) compared with no difficulty. The OR for waking in the middle of the night or early morning less than once per week was 4.61 (0.85–24.9), 1–2 times per week was 1.49 (0.18–12.6), and ≥3 times per week was 1.35 (0.09–20.5) compared with not waking in the middle of the night or early morning. For the same adjustment variable, adjusted ORs of “fairly bad” and “very bad” compared with “fairly good or very good” sleep quality were 2.55 (95% CI, 0.56–11.7) and 8.76 (95% CI, 0.47–162), respectively.

Additional analysis was conducted to investigate interactions in variables. Relationship problems in the workplace is a two-category variable: no and yes. Fatigue on waking was divided into two categories: “less than once per month” and “once or more per month”. The global score of PSQI was divided into two categories: “5 or less (no sleep disturbance)” and “6 or more (sleep disturbance)”. Multivariate analysis was conducted with the three two-category variables included simultaneously in the model (Model 3) (not shown in Table 1). The explanatory variables of Model 3 other than the three variables (relationship problems in the workplace, fatigue on waking, PSQI score) were the same as those used in Model 1, except for relationship problems in the workplace and fatigue on waking. In Model 3, the ORs were as follows: relationship problems in the workplace (3.36 [0.78–14.4]), fatigue on waking once or more per month (6.07 [1.02–36.1]), and PSQI score ≥ 6 (2.72 [0.61–12.1]). Only fatigue on waking once or more per month showed a significant association with DSI. The most significant factor in predicting suicidal ideation was fatigue on waking in this model. Model 3 was employed to calculate the interaction of the three variables. No significant interactions were observed for relationship problems in the workplace and fatigue on waking (*p* = 0.836), fatigue on waking and PSQI score (*p* = 0.576), or relationship problems in the workplace and PSQI score (*p* = 0.904).

Sensitivity and specificity, positive and negative predictive values, and likelihood ratios for DSI were calculated (Table 2). The lowest LR− (0.171) was observed for “relationship problems in the workplace (RPW), PSQI score ≥ 6 (PS6), or fatigue on waking once or more per month (FWM).” High LR+ values (57.54, 11.99, and ∞) were observed for “relationship problems in the workplace (RPW) and PSQI score ≥ 10 (PS10),” “relationship problems in the workplace (RPW), PSQI score ≥ 6 (PS6), and fatigue on waking once or more per month (FWM),” and “relationship problems in the workplace (RPW), PSQI score ≥ 10 (PS10), and fatigue on waking once or more per week (FWW),” respectively. For subjects who did not have “relationship problems in the workplace, PSQI score ≥ 6, or fatigue on waking once or more per month (RPW, PS6, or FWM)” the post-inquiry probability was 0.59% with a negative predictive value of 99.41%. Table 2 displays the value as 99.4 because 99.41 was rounded to a single decimal place. “Relationship problems in the workplace, PSQI score ≥ 6, and fatigue on waking once or more per month (RPW, PS6 and FWM)” and “relationship problems in the workplace and PSQI score ≥ 10 (RPW and PS10)” showed post-inquiry probabilities of 29.4% and 66.7%, respectively. Two subjects had relationship problems in the workplace (RPW), PSQI score ≥ 10 (PS10), and fatigue on waking once or more per week (FWW) at enrollment. Both of these subjects developed DSI.

Weak but statistically significant correlations were observed between fatigue on waking and rating of sleep quality (Spearman’s correlation coefficient of r = 0.286, *p* < 0.0001), fatigue on waking and total PSQI score (r = 0.267, *p* < 0.0001), and fatigue on waking and difficulty maintaining enough enthusiasm to get things done (one of the items included in the PSQI) (r = 0.220, *p* < 0.0001). Thus, the correlated variables of fatigue on waking and total PSQI score were not included simultaneously as explanatory variables in the calculations shown in Table 1.

The calculated statistical powers were 0.797 for relationship problems in the workplace, 0.813 for fatigue on waking, 0.780 for PSQI score (≤5 and ≥6), 0.797 for PSQI score (≤6 and ≥7), and 0.635 for PSQI score (≤9 and ≥10). Only fatigue on waking showed a higher power than 0.8, but all calculated values were higher than 0.5 [60].

## 4. Discussion

Because the number of subjects was small, the results of the present investigation should be interpreted carefully. Only 13 subjects had DSI. More studies using different methods with larger sample sizes in different places are needed to confirm our findings.

The three predictive factors for DSI identified in this study were fatigue on waking, poor sleep, and relationship problems in the workplace. Significantly increased ORs were observed for fatigue on waking (ORs: 7.90 and 6.79), total PSQI score of ≥10 (OR: 8.19), and relationship problems in the workplace (ORs: 4.24 and 4.84). These predictive factors may be useful for predicting DSI in a primary care setting at 6 months from the initial medical consultation. These findings may also help physicians to decide, within a period of 6 months, whether to initiate treatment or refer the patient to a mental health specialist.

The current investigation involved several unique aspects. First, the subjects were 387 new primary care patients without depression. Second, 13 newly identified cases (i.e., incident cases) of DSI were observed at 6 months after an initial medical consultation. Third, the 6-month follow-up was shorter than in previous investigations that followed subjects for years to decades [26,61,62,63]. Periods of several years of observation with no treatment are excessively long and are impractical in clinical practice.

Combinations of these three conditions—fatigue on waking, poor sleep, and relationship problems in the workplace—showed high LRs+ of 11.99 to ∞ and the lowest LR− of 0.171. High LR+ and low LR− indicate specific questions and sensitive questions, respectively. Specific combinations of symptoms can be used in the diagnostic process, and sensitive combinations of the symptoms may be used to rule out a diagnosis. In general, tests with LRs further from 1.0 are associated with fewer false positives and fewer false negatives [58]. The most sensitive combination of “relationship problems in the workplace, PSQI score ≥ 6, or fatigue on waking once or more per month (RPW, PS6, or FWM)” had the lowest LR− of 0.171. Subjects who did not have any of these three conditions had a post-inquiry probability of DSI of 0.59%. This was clearly lower than the pre-inquiry probability of 3.36% (calculated by dividing the number of patients with DSI [13 subjects] by the total number of subjects [387 subjects]). The specific combinations of “relationship problems in the workplace, PSQI score ≥ 6, and fatigue on waking once or more per month (RPW, PS6, and FWM)” and “relationship problems in the workplace and PSQI score ≥ 10 (RPW and PS10)” showed post-inquiry probabilities of 29.4% and 66.7%, respectively; these were substantially higher than the pre-inquiry probability of 3.36%. If physicians took the time to inquire about fatigue on waking and relationship problems in the workplace, and determined the patient’s total PSQI score, they might be able to more quickly evaluate the probability of DSI within 6 months. Even during monthly or weekly follow-ups, these three factors may help to predict DSI, enabling physicians to initiate timely treatment for depression or initiate a referral to a mental health specialist.

Riihimäki et al., reported that one-tenth of primary care patients with depressive disorders attempted suicide within 5 years. They also found that a current major depressive episode was a significant independent risk factor (hazard ratio 33.5 [95% CI: 3.6–309.7]) [26]. Barraclough et al., investigated hundreds of suicides and found that 70% of deceased subjects had depression [64]. Chynoweth et al., conducted a similar investigation of 135 suicides and found that 55% had a depressive disorder [65]. Because of the increased risk of suicide among depressive patients, we explored predictive factors for DSI.

Two meta-analyses examined studies on insomnia and suicidal behavior in populations other than primary care patients [27,28]. Pigeon et al., reported that sleep disturbances showed significant associations with suicidal ideation, suicide attempts, and suicide (OR: 1.86–2.01) [28]. Malik et al., reported a significant association between sleep disturbances and suicidal behaviors among depressive patients (OR: 3.05 [2.07–4.48], *p* < 0.001) [27]. However, Skapinakis et al., reported that some previous studies may have overestimated the importance of sleep disturbances as an independent risk factor for depression [66]. They explained that a strong cross-sectional association is compatible with sleep disturbances as a prodromal or residual symptom of depression.

Owusu et al., reported an association of sleep characteristics with suicidal ideation and suicide attempt among adults aged 50 and older with depressive symptoms in low-and middle-income countries [67]. They showed that subjects with poor/very poor sleep quality had greater odds of suicidal ideation. Subjects with moderate and severe/extreme insomnia symptoms had greater odds of suicidal ideation and suicide attempts. These findings are consistent with those of the present investigation. Severe insomnia with PSQI score ≥ 10 was significantly associated with DSI. However, sleep quality was not associated with DSI in multivariate analyses in the present study, as mentioned in the Results section. This may be partly due to the small sample size of the present investigation.

In the current study, subjects with a depressive history or with depression or suicidal ideation at enrollment (at the initial medical consultation), who might not be at risk for newly identified DSI after 6 months, were excluded, because predictive factors of newly identified cases may differ from those of recurrent or persistent cases [68,69]. Subjects who had poor sleep at the initial medical consultation because of depressive symptoms were excluded from this study. Thus, the cross-sectional effect of poor sleep (i.e., PSQI global score) and DSI was minimized. This may explain why waking in the middle of the night or early morning, difficulty falling asleep within 30 min, quality of sleep, and average length of sleep in the past month did not show a significant association with DSI in the present study.

Chronic physical illness was found to be a predictor of suicidal behavior in a previous investigation [70]. Additionally, associations between depression and physical illnesses were reported previously [71,72,73]. However, we did not find an association between DSI and physical illnesses (e.g., circulatory illnesses, diabetes, or stroke) in the current study, possibly because patients who required periodical consultation at a medical institution for severe physical illnesses were unlikely to have been included in our investigation. Enrolled subjects were new patients who visited one of three primary care clinics for an initial medical consultation, not for regular consultation.

We found that relationship problems in the workplace showed a significant association with DSI. Previous studies have reported similar results. A cross-sectional study showed that interpersonal conflict was significantly associated with depression, particularly among highly educated males [35]. Another cross-sectional study reported that poor social support was independently associated with suicidal ideation (OR: 3.1 [95% CI: 2.6–3.7]) [74]. In that study, poor social support showed a relatively high population attributable fraction of 38% for suicidal ideation [74]. A study of two nationally representative samples (one from England and one from the United States) also reported a significant negative association between social support and suicide attempts (OR = 0.68–0.93) [75]. The present findings suggest that inquiries should focus not only on an individual’s health problems but also on their relationship problems to prevent DSI.

Associations between social functioning and depression have been reported [76,77]. In this present study, ORs for DSI for similar items included in the social functioning scale [78], such as occupation, type of employment, marital status, family member, family member living together, outpatient visits to other clinic(s) or hospital(s), and feeling of isolation from other family members, were calculated (univariate analysis). In the univariate analyses, only feeling of isolation from other family members (≥20.0 mm in visual analogue scale) showed a significant OR (3.67 [1.04–13.0]). Therefore, a feeling of isolation from other family members was included in the final model shown in Table 1. Because of the limited sample size of the present investigation, a feeling of isolation from other family members might not show a significant OR in the multivariate analysis.

According to the American Psychiatric Association’s Diagnostic and Statistical Manual of Mental Disorders Fifth Edition (DSM-5), to be classified as depressive, patients must be experiencing five or more depressive symptoms most of the day, nearly every day, during the same 2-week period, and at least one of the symptoms must be either depressed mood or loss of interest/pleasure. Additionally, depressive symptoms must cause clinically significant distress or impairment in social, occupational, or other important areas of functioning, and the symptoms must not be caused by substance abuse or another medical condition. Although the diagnosis of depression in this study did not fully correspond to the DSM-5 [79], we succeeded in eliminating the observer bias that can occur when multiple interviewers make diagnoses [80]. Our use of the SDS and POMS, which are widely employed for clinical and research purposes, enabled us to assess all subjects using consistent criteria without observer bias. Observer bias could have caused unexpected over- or under-estimation of the association between predictive factors and DSI [69]. Additionally, patients with psychosis or dementia, whose ability to report their condition may be impaired, were not included in this study [80].

The current study involved several limitations. First, the number of enrolled subjects was small because we aimed to obtain complete datasets despite limited resources. Power analyses were difficult to conduct before the data collection of the current study. The frequency of newly identified cases of DSI after several months, relationships in the workplace, and fatigue on waking among non-depressed primary care patients were not investigated, as far as we know. Additionally, this was not a nationwide multicenter study; it was conducted at only three internal medicine clinics located in Fuji City, which is an industrial city with a population of 250,000. Because most enrolled subjects (99.5%) were inhabitants of Fuji City and its surrounds, differences in subject characteristics related to place of residence were minimized. Another limitation is that we did not identify whether fatigue on waking and total PSQI score represented prodromal symptoms or causes of DSI. Finally, suicidal ideation was only assessed using self-administered questionnaires. One item in the SDS (“I feel that others would be better off if I were dead”), which was used to assess suicidal ideation, may reflect suicidal thought rather than suicide attempts (suicidal behaviors) [81]. According to Kessler, approximately 90% of unplanned suicide attempts and 60% of planned first suicide attempts occur within 1 year of the onset of ideation [82]. Thus, it is reasonable to investigate suicidal ideation because suicidal ideation is the first layer of the pyramid of occurrence of suicidal ideation and non-fatal and fatal behavior [81].

This study focuses on the three identified predictive factors that may assist doctors in detecting patients who are at risk of suicidal ideation. Managing depression symptoms, recognizing depression, and beginning treatment are still important because of the increased risk of suicide among depressive patients, as mentioned in the Introduction and Discussion sections. In a time-constrained busy primary care setting, depression is frequently overlooked. Thus, not only physicians but also other health care professionals need to assess and identify the signs and symptoms of depression to prevent suicidal ideation and behaviors.

The present results must be interpreted carefully, and the findings may not be generalizable beyond middle-aged primary care patients. The results in the current study may also not be generalizable to the period after 2019. The COVID-19 pandemic has altered social, working, and home environments [83,84]. According to the Organisation for Economic Co-operation and Development (OECD) Health at a Glance 2021, the prevalence of anxiety and depression were more than double the levels observed before the COVID-19 pandemic in most countries, including Japan, with available data [85]; however, the restrictions of the pandemic on society have eased as of March 2023 in Japan. The effects of the pandemic may influence the prevalence of mental disease for several years beyond the peak of the pandemic.

Further studies using different methods in different locations will be needed to clarify the predictive and risk factors for DSI in the primary care setting.

## 5. Conclusions

Signs of DSI often go undetected in primary care facilities, particularly during initial consultations in a busy clinical practice. The present results revealed that inquiring about the frequency of fatigue on waking, sleep status and relationship problems in the workplace may be helpful for predicting DSI in primary care settings. Because the sample size in this investigation was small, further studies with larger samples are needed to confirm our findings.

## Figures and Tables

**Table 1:** Odds ratios for depression with suicidal ideation (DSI) and characteristics of subjects with and without DSI among primary care patients aged 35–64 years.

	Subjects with DSI		Subjects without DSI		Crude		Model 1 ^†^		Model 2 ^‡^	
	(*n* = 13)		(*n* = 374)		OR (95% CI)	*p* Value	OR (95% CI)	*p* Value	OR (95% CI)	*p* Value
	*n* (%)		*n* (%)							
Sex										
Male	6	(46.2)	182	(48.7)	1.00		1.00		1.00	
Female	7	(53.9)	192	(51.3)	1.11 (0.37–3.35)	0.859	2.43 (0.46–12.9)	0.296	2.64 (0.46–15.1)	0.277
Age (median, range)	52.0 (40.0–64.3) 48.9 (35.1–64.4)									
35–44 (years)	3	(23.1)	145	(38.8)	1.00		1.00		1.00	
45–54	8	(61.5)	120	(32.1)	3.22 (0.84–12.4)	0.089	5.64 (0.96–33.3)	0.056	4.19 (0.70–25.3)	0.118
55–64	2	(15.4)	109	(29.1)	0.89 (0.15–5.40)	0.896	2.52 (0.28–23.0)	0.414	1.20 (0.14–10.3)	0.867
					(*p* for trend 0.933)		(*p* for trend 0.288)		(*p* for trend 0.810)	
Regular exercise										
Less than once per month	12	(92.3)	244	(65.2)	1.00		1.00		1.00	
Once or more per month	1	(7.7)	130	(34.8)	0.16 (0.02–1.22)	0.076	0.17 (0.02–1.76)	0.136	0.19 (0.02–1.82)	0.148
Problems involving care of an ill or elderly family member during the previous 6 months										
No	10	(76.9)	345	(92.3)	1.00		1.00		1.00	
Yes	3	(23.1)	29	(7.8)	3.57 (0.93–13.7)	0.064	4.44 (0.76–25.9)	0.098	5.61 (0.88–35.6)	0.067
Death of a family member during the previous 6 months										
No	11	(84.6)	361	(96.5)	1.00		1.00		1.00	
Yes	2	(15.4)	13	(3.5)	5.05 (1.01–25.1)	0.048	3.23 (0.38–27.8)	0.286	3.07 (0.35–26.8)	0.310
Relationship problems in the workplace										
No	7	(53.9)	324	(86.6)	1.00		1.00		1.00	
Yes	6	(46.2)	50	(13.4)	5.56 (1.79–17.2)	0.003	4.24 (1.00–17.9)	0.049	4.84 (1.14–20.6)	0.033
Cigarette smoking										
Never or former	6	(46.2)	286	(76.5)	1.00		1.00		1.00	
Current	7	(53.9)	88	(23.5)	3.79 (1.24–11.6)	0.019	7.48 (1.27–44.2)	0.027	3.42 (0.65–17.9)	0.146
History of psychiatric disorder excluding dementia in parent(s) or child (children)										
None	10	(76.9)	350	(93.6)	1.00		1.00		1.00	
Parent(s) or child (children) with psychiatric illness(es)	3	(23.1)	24	(6.4)	4.38 (1.13–17.0)	0.033	4.94 (0.85–28.6)	0.074	3.03 (0.45–20.2)	0.253
Educational background										
Junior high school	1	(7.7)	15	(4.0)	1.74 (0.20–15.1)	0.614	1.01 (0.03–31.2)	0.998	1.61 (0.06–42.0)	0.776
High school	7	(53.9)	183	(48.9)	1.00		1.00		1.00	
Technical school, junior college, or higher vocational school	2	(15.4)	88	(23.5)	0.59 (0.12–2.92)	0.522	0.34 (0.04–2.59)	0.296	0.24 (0.02–2.32)	0.216
University or graduate school	3	(23.1)	88	(23.5)	0.89 (0.23–3.53)	0.870	0.93 (0.15–5.84)	0.941	0.77 (0.12–4.72)	0.773
Mean alcohol consumption per day										
non-drinker	2	(15.4)	147	(39.3)	1.00		1.00		1.00	
≤34 (g)	10	(76.9)	186	(49.7)	3.95 (0.85–18.3)	0.079	4.71 (0.77–28.7)	0.093	4.74 (0.71–31.8)	0.109
>34	1	(7.7)	41	(11.0)	1.79 (0.16–20.3)	0.637	3.50 (0.17–70.3)	0.413	3.51 (0.18–67.4)	0.406
					(*p* for trend 0.261)		(*p* for trend 0.169)		(*p* for trend 0.209)	
Feeling of isolation from other family members (visual analogue scale)										
<10.0 (mm)	7	(53.9)	302	(80.8)	1.00		1.00		1.00	
10.0–19.9	2	(15.4)	25	(6.7)	3.45 (0.68–17.5)	0.135	2.92 (0.33–25.7)	0.335	3.35 (0.42–26.5)	0.252
≥20.0	4	(30.8)	47	(12.6)	3.67 (1.04–13.0)	0.044	1.91 (0.36–10.1)	0.447	2.98 (0.54–16.4)	0.210
					(*p* for trend 0.030)		(*p* for trend 0.320)		(*p* for trend 0.145)	
Fatigue on waking										
Less than once per month	3	(23.1)	240	(64.2)	1.00		1.00			
Once or more per month but less than once per week	4	(30.8)	43	(11.5)	7.44 (1.61–34.4)	0.010	7.90 (1.06–58.7)	0.043		
Once or more per week	6	(46.2)	91	(24.3)	5.28 (1.29–21.5)	0.021	6.79 (1.02–45.1)	0.048		
					(*p* for trend 0.015)		(*p* for trend 0.051)			
Global score on Pittsburgh Sleep Quality Index										
≤5	4	(30.8)	259	(69.3)	1.00				1.00	
6	2	(15.4)	47	(12.6)	2.76 (0.49–15.5)	0.250			2.15 (0.28–16.5)	0.463
7–9	5	(38.5)	58	(15.5)	5.58 (1.45–21.4)	0.012			3.22 (0.56–18.7)	0.193
≥10	2	(15.4)	10	(2.7)	13.0 (2.12–79.2)	0.006			8.19 (1.05–63.8)	0.045
					(*p* for trend 0.001)				(*p* for trend 0.038)	

DSI: depression with suicidal ideation; OR: odds ratio; CI: confidence interval. ^†^ Explanatory variables: sex, age, regular exercise habits, problems involving care of the family, death of a family member, relationship problems in the workplace, cigarette smoking, history of psychiatric disorder excluding dementia in parent(s) or child (children), educational background, mean alcohol consumption per day, feeling of isolation from family members, and fatigue on waking. ^‡^ Explanatory variables: sex, age, regular exercise habits, problems involving care of the family, death of a family member, relationship problems in the workplace, cigarette smoking, history of psychiatric disorder excluding dementia in parent(s) or child (children), educational background, mean alcohol consumption per day, feeling of isolation from family members, and Pittsburgh Sleep Quality Index global score.

**Table 2:** Sensitivity, specificity, positive and negative predictive values, and likelihood ratios for depression with suicidal ideation (DSI) among primary care patients aged 35–64 years.

Condition	Sensitivity (%) (95% CI)	Specificity (%) (95% CI)	Positive Predictive Value (%) (95% CI)	Negative Predictive Value (%) (95% CI)	Positive Likelihood Ratio (95% CI)	Negative Likelihood Ratio (95% CI)	DSI/Number of Subjects with Condition (s)
Relationship problems in the workplace, Pittsburgh Sleep Quality Index score ≥ 10, and fatigue on waking once or more per week (RPW, PS10, and FWW)	15.4	100	100	97.1	∞	0.846	2/2
	(1.9–45.5)	(99.0–100)	(15.8–100)	(95.0–98.6)		(0.671–1.067)	
Relationship problems in the workplace and Pittsburgh Sleep Quality Index score ≥ 10 (RPW and PS10)	15.4	99.7	66.7	97.1	57.54	0.848	2/3
	(1.9–45.5)	(98.5–99.99)	(9.4–99.2)	(94.9–98.6)	(5.565–594.9)	(0.673–1.070)	
Relationship problems in the workplace, Pittsburgh Sleep Quality Index score ≥ 6, and fatigue on waking once or more per month (RPW, PS6, and FWM)	38.5	96.8	29.4	97.8	11.99	0.636	5/17
	(13.9–68.4)	(94.5–98.3)	(10.3–56.0)	(95.8–99.1)	(4.949–29.04)	(0.414–0.978)	
Relationship problems in the workplace and Pittsburgh Sleep Quality Index score ≥ 6 (RPW and PS6)	46.2	94.4	22.2	98.1	8.220	0.570	6/27
	(19.2–74.9)	(91.5–96.5)	(8.6–42.3)	(96.0–99.2)	(4.004–16.88)	(0.345–0.944)	
Relationship problems in the workplace and fatigue on waking once or more per week (RPW and FWW)	38.5	95.2	21.7	97.8	7.991	0.646	5/23
	(13.9–68.4)	(92.5–97.1)	(7.5–43.7)	(95.7–99.1)	(3.512–18.18)	(0.420–0.994)	
Pittsburgh Sleep Quality Index score ≥ 10 (PS10)	15.4	97.3	16.7	97.1	5.754	0.869	2/12
	(1.9–45.5)	(95.1–98.7)	(2.1–48.4)	(94.8–98.5)	(1.399–23.66)	(0.689–1.097)	
Relationship problems in the workplace and fatigue on waking once or more per month (RPW and FWM)	38.5	93.3	16.7	97.8	5.754	0.659	5/30
	(13.9–68.4)	(90.3–95.6)	(5.6–34.7)	(95.6–99.0)	(2.624–12.62)	(0.429–1.014)	
Relationship problems in the workplace (RPW)	46.2	86.6	10.7	97.9	3.452	0.622	6/56
	(19.2–74.9)	(82.8–89.9)	(4.0–21.9)	(95.7–99.2)	(1.818–6.556)	(0.375–1.030)	
Relationship problems in the workplace or Pittsburgh Sleep Quality Index score ≥ 10 (RPW or PS10)	46.2	84.2	9.2	97.8	2.926	0.639	6/65
	(19.2–74.9)	(80.1–87.8)	(3.5–19.0)	(95.6–99.1)	(1.555–5.505)	(0.386–1.060)	
Pittsburgh Sleep Quality Index score ≥ 6 (PS6)	69.2	69.3	7.3	98.5	2.252	0.444	9/124
	(38.6–90.9)	(64.3–73.9)	(3.4–13.3)	(96.2–99.6)	(1.520–3.336)	(0.196–1.007)	
Fatigue on waking once or more per month (FWM)	76.9	64.2	6.9	98.8	2.147	0.360	10/144
	(46.2–95.0)	(59.1–69.0)	(3.4–12.4)	(96.4–99.7)	(1.548–2.978)	(0.133–0.973)	
Relationship problems in the workplace or fatigue on waking once or more per month (RPW or FWM)	84.6	57.5	6.5	99.1	1.990	0.268	11/170
	(54.6–98.1)	(52.3–62.6)	(3.3–11.3)	(96.7–99.9)	(1.535–2.581)	(0.075–0.960)	
Fatigue on waking once or more per week (FWW)	46.2	75.7	6.2	97.6	1.897	0.712	6/97
	(19.2–74.9)	(71.0–79.9)	(2.3–13.0)	(95.1–99.0)	(1.027–3.504)	(0.429–1.181)	
Relationship problems in the workplace or Pittsburgh Sleep Quality Index score ≥ 6 (RPW or PQ6)	69.2	61.5	5.9	98.3	1.798	0.500	9/153
	(38.6–90.9)	(56.4–66.5)	(2.7–10.9)	(95.7–99.5)	(1.224–2.641)	(0.221–1.135)	
Relationship problems in the workplace, Pittsburgh Sleep Quality Index score ≥ 6, or fatigue on waking once or more per month (RPW, PS6, or FWM)	92.3	44.9	5.5	99.4	1.676	0.171	12/218
	(64.0–99.8)	(39.8–50.1)	(2.9–9.4)	(96.8–99.99)	(1.397–2.010)	(0.026–1.130)	
Relationship problems in the workplace or fatigue on waking once or more per week (RPW or FWW)	53.9	67.1	5.4	97.7	1.637	0.688	7/130
	(25.1–80.8)	(62.1–71.9)	(2.2–10.8)	(95.0–99.1)	(0.970–2.764)	(0.381–1.242)	
Relationship problems in the workplace, Pittsburgh Sleep Quality Index score ≥ 10, or fatigue on waking once or more per week (RPW, PQ10, or FWW)	53.9	65.0	5.1	97.6	1.537	0.710	7/138
	(25.1–80.8)	(59.9–69.8)	(2.1–10.2)	(94.8–99.1)	(0.912–2.591)	(0.393–1.284)	

Note: Specificity is 100% thus the positive likelihood ratio is showed as infinity “∞”. DSI: depression with suicidal ideation; CI: confidence interval. RPW: relationship problems in the workplace. PS10: Pittsburgh Sleep Quality Index score ≥ 10, PS6: Pittsburgh Sleep Quality Index score ≥ 6. FWM: fatigue on waking once or more per month, FWW: fatigue on waking once or more per week.

## Data Availability

Restrictions apply to the availability of these data. Data are available from the corresponding authors with the permission of Kurume University. The data are not publicly available because of privacy concerns, and ethical and legal restrictions.
